# Volatile organic compounds emitted by *Trichoderma* species mediate plant growth

**DOI:** 10.1186/s40694-016-0025-7

**Published:** 2016-09-29

**Authors:** Samantha Lee, Melanie Yap, Gregory Behringer, Richard Hung, Joan W. Bennett

**Affiliations:** 1 Department of Plant Biology and Pathology, Rutgers, The State University of New Jersey, New Brunswick, NJ USA; 2 Department of Biochemistry, Microbiology and Molecular Biology, Pennsylvania State University, 309 Life Sciences Building, State College, PA 16803 USA; 3 Chemistry Program, Division of Science, New York University Abu Dhabi, PO Box 129188, Abu Dhabi, United Arab Emirates; 4 Biology Department, Kean University, 1000 Morris Ave., Union, NJ 07083 USA

**Keywords:** Volatile organic compounds, *Trichoderma*, Plant growth, *Arabidopsis thaliana*, *Solanum lycopersicum*, Gas chromatography–mass spectrometry, Plant–microbe interactions

## Abstract

**Background:**

Many *Trichoderma* species are applied as biofungicides and biofertilizers to agricultural soils to enhance crop growth. These filamentous fungi have the ability to reduce plant diseases and promote plant growth and productivity through overlapping modes of action including induced systemic resistance, antibiosis, enhanced nutrient efficiency, and myco-parasitism. *Trichoderma* species are prolific producers of many small metabolites with antifungal, antibacterial, and anticancer properties. Volatile metabolites of *Trichoderma* also have the ability to induce resistance to plant pathogens leading to improved plant health. In this study, *Arabidopsis* plants were exposed to mixtures of volatile organic compounds (VOCs) emitted by growing cultures of *Trichoderma* from 20 strains, representing 11 different *Trichoderma* species.

**Results:**

We identified nine *Trichoderma* strains that produced plant growth promoting VOCs. Exposure to mixtures of VOCs emitted by these strains increased plant biomass (37.1–41.6 %) and chlorophyll content (82.5–89.3 %). *Trichoderma* volatile-mediated changes in plant growth were strain- and species-specific. VOCs emitted by *T*. *pseudokoningii* (CBS 130756) were associated with the greatest *Arabidopsis* growth promotion. One strain, *T. atroviride* (CBS 01-209), in our screen decreased growth (50.5 %) and chlorophyll production (13.1 %). Similarly, tomatoes exposed to VOCs from *T. viride* (BBA 70239) showed a significant increase in plant biomass (>99 %), larger plant size, and significant development of lateral roots. We also observed that the tomato plant growths were dependent on the duration of the volatile exposure. A GC–MS analysis of VOCs from *Trichoderma* strains identified more than 141 unique compounds including several unknown sesquiterpenes, diterpenes, and tetraterpenes.

**Conclusions:**

Plants grown in the presence of fungal VOCs emitted by different species and strains of *Trichoderma* exhibited a range of effects. This study demonstrates that the blend of volatiles produced by actively growing fungi and volatile exposure time in plant development both influence the outcome of volatile-mediated interactions. Only some of our growth promoting strains produced microbial VOCs known to enhance plant growth. Compounds such as 6-pentyl-2*H*-pyran-2-one were not common to all promoting strains. We found that biostimulatory strains tended to have a larger number of complex terpenes which may explain the variation in growth induced by different *Trichoderma* strains.

## Background

The genus *Trichoderma* is one of the most widely researched genera of filamentous fungi with numerous applications in agriculture, industry, and the environment [1, 2]. Several *Trichoderma* species have the ability to reduce plant diseases and promote plant growth and productivity by utilizing overlapping modes of action including induced systemic resistance [3, 4], antibiosis [5], enhanced nutrient efficiency [6], and myco-parasitism [7, 8]. In agriculture, *Trichoderma* species are robust biological control agents and are often added to soils to increase crop yields and control soil-borne pathogen worldwide. It has been estimated that in India alone, more than 250 *Trichoderma*-based formulations are sold commercially [9–11]. Moreover, since *Trichoderma* species possess innate resistance to many chemicals used in agriculture such as fungicides, they are readily integrated into pest management practices [12].

*Trichoderma* species are prolific producers of many small metabolites with medical and agricultural significance [13, 14]. Secondary metabolites such as peptaibols and polyketides exhibit antifungal, antibacterial, and anticancer properties; induce resistance to plant pathogens; or serve as toxins [14, 15]. Volatile metabolites, also known as volatile organic compounds (VOCs), have low molecular mass, high vapor pressure (>0.01 kPa), low boiling point, and low polarity [16]. They are chemically diverse and include hydrocarbons, aromatics, amines, thiols, and terpenes [17, 18]. Relatively little is known about the metabolic origin of these compounds in fungi but in plants, similar volatiles are produced as breakdown products of fatty acids, others are biotransformation products of molecules produced in central metabolism while the terpenes are secondary metabolites [19, 20].

Furthermore, there are limited studies focused on *Trichoderma* VOCs and their impact on plant growth. For example, the coconut odor volatile, 6-pentyl-2*H*-pyran-2-one (6PP), is one of the earliest volatile compounds to be characterized from *Trichoderma*; however, it was studied for its use as a “nature identical flavouring” in the food industry [21, 22]. It was not until 2008 that the effects of 6PP on plant growth and disease symptoms were examined. Adding 6PP (0.166–1 mg/l) to plant growth media or directly applying a 6PP solution to plant leaves induced growth promotion and reduced disease symptoms [23]. Recently, our laboratory has reported the ability of mixtures of VOCs from *Trichoderma viride* to stimulate plant growth in the absence of pathogen attack or physical contact with the plant. *Arabidopsis thaliana* exposed to *T. viride*-derived VOCs had increased plant size, fresh weight, chlorophyll, root growth, and number of flowers even in the absence of pathogen threat [24]. The volatile-mediated plant growth promotion was dependent on *Trichoderma* species, culture, developmental stage of the plants, and duration of the exposure [24, 25].

Many species of *Trichoderma* are useful biocontrol organisms known to enhance crop yields when added to soils. Our recent studies demonstrate that *T. viride* and *T. atroviride* may be able to increase plant vigor by emitting volatile blends. The aims of this study were to demonstrate that *Trichoderma* VOCs are a major factor in plant growth promotion, identify potential strains, and identify compounds or combination of compounds that could be directly applied to induce plant growth. To this end, we assessed *Arabidopsis* growth and development when plants were grown in a shared atmosphere with VOCs emitted by 20 *Trichoderma* strains from 11 different species. We also replicated the VOC-induced growth enhancement effects with the economically important crop, *Solanum lycopersicum* (tomato). Finally, we characterized the volatile profiles emitted by strains using gas chromatography–mass spectrometry (GC–MS) analysis and assessed trends shared among positively acting *Trichoderma* strains.

## Methods

### Cultivation of fungal strains

*Trichoderma* strains were obtained from Dr. Amy Y. Rossman at USDA-ARS, Beltsville, MD. See Table 1 for strain number, as well as the original location and substrates from which the strains were isolated. Cultures were maintained on potato dextrose agar (PDA) or malt extract agar (MEA) (Difco) at 27 ± 2 °C in the dark with >80 % humidity. For the volatile-exposure bioassay, the fungus was grown in a 35 × 10 mm Petri dish containing 4 ml of MEA and incubated for 5 days at 27 ± 2 °C.

**Table 1:** *Trichoderma* strains screened for volatile-induced growth promotion

Strain #	*Trichoderma* species	Strain code	Location	Source
1	*T. aggressivum*	DAOM222156	ON, Canada	Mushroom casing
2	*T. aggressivum*	IMI 393970	PA, USA	Mushroom compost
3	*T. asperellum*	CBS 433.97	Beltsville, MD, USA	Soil, sclerotia buried in sesame plot
4	*T. asperellum*	GJS 02-65	Douala, Loum, Cameroon	Soil, *Xanthosoma sagittifolium* roots and soil
5	*T. atroviride*	CBS 351.93	NC, USA	Soil, forest
6	*T. atroviride*	GJS 01-209	Cameroon	Palm
7	*T. atroviride*	JWB	New Orleans, LA, USA	Building, Hurricane Katrina damaged
8	*T. brevicompactum*	CBS 109720	NY, USA	Soil, under Helianthus
9	*T. harzianum*	CBS 226.95	United Kingdom	Soil
10	*T. harzianum*	CBS 227.95	United Kingdom	Soil
11	*T. inhamantum*	CBS 273.78	Colombia	Soil, maize field
12	*H. koningii*	CBS 989.97	MD, USA	Decorticated wood (*T. koningii* type specimen)
13	*T. longibrachiatum*	CBS 118642	Mexico	Soil
14	*T. longibrachiatum*	TR97	OH, USA	Soil
15	*T. pseudokoningii*	CBS 480.91	Australia	Wood, decayed
16	*T. pseudokoningii*	CBS 130756	Australia	Wood, decorticated
17	*T. stromaticum*	GJS 00-127	Bahia, Brazil	Theobroma cacao, pod
18	*T. virens*	DAOM167651	GA, USA	Soil, cultivated
19	*T. viride*	BBA 70239	Denmark	Building, water damaged
20	*T. viride*	GJS 04-379	Brazil	Soil

Abbreviations of culture collections and collectors as follows: *BBA* Biologisches Bundesanstalt, Berlin, Germany; *CBS* Centraalbureau voor Schimmelcultures, Utrecht, The Netherlands; *DAOM* Canadian Collection of Fungal Cultures, Ottawa, Canada; *GJS* Gary J. Samuels collection (Culture collection of the United States Department of Agriculture, Systematic Botany and Mycology Lab, Beltsville, MD, USA); *IMI* International Mycological Institute (New Zealand); *JWB* Joan W. Bennett collection (Rutgers, New Brunswick, NJ, USA); *TR* Earl Nelson collection (USDA-ARS, Beltsville)

### Plant materials and growth conditions

*Arabidopsis thaliana* seeds (ecotype Columbia-7) were obtained from the *Arabidopsis* Biological Resource Center (Columbus, OH). The seeds were surface-sterilized in 95 % ethanol for 30 s followed by a 20 % bleach solution for 30 min with constant agitation. Five surface-sterilized seeds were sown onto a 100 × 15 mm partitioned Petri dish (also known as split or I-plate) or 60 × 15 mm Petri dish containing Murashige and Skoog (MS) medium (pH 5.7) with vitamins, 3 % sucrose, and 0.03 % phytagel (Phytotechnology Laboratories, KS). Seeds were stratified at 4 °C for 3 days prior to volatile exposure.

Seeds of tomato (*Solanum lycopersicum* L. cv. Ponderosa) were purchased commercially. Seeds were surface sterilized with 70 % ethanol for 30 s and a 15 % bleach solution for 20 min with constant agitation. Prior to the exposure assay, two surface-sterilized seeds were sown onto a 473 ml volume sterile culture vessel (SteriCon, PhytoTechnology Laboratories, KS) containing 100 ml of MS media (1 % sucrose, 0.03 % phytagel, and pH 5.7).

### Plant-*Trichoderma* volatile-exposure bioassay

Exposures of *Arabidopsis* plants to *Trichoderma* VOCs were performed using a double plate-within-a-plate system according to previously described methods [25]. Briefly, a small Petri plate (35 × 10 mm) containing sporulating *Trichoderma* grown on MEA was placed into a larger partitioned Petri dish (100 × 15 mm) containing five stratified *A. thaliana* seeds. They were grown together in a shared atmosphere in a growth chamber with a 16 h photoperiod at 23 ± 1 °C, 45 % relative humidity, and 180 µmol m^−2^ s^−1^ light for 14 days. For controls, plants were exposed to the fungal growth medium alone.

To expose tomato seeds, a Petri plate (35 × 10 mm) containing the *Trichoderma* culture was placed inside a sterile foil container (50 ml volume); together they were then placed inside the culture vessel containing sterilized seeds. Tomato seeds were germinated in the presence of *Trichoderma* VOCs and grown with one another in a shared atmosphere in a growth chamber with a 16-h photoperiod at 25 ± 1 °C for 21 days. At the end of the VOC exposure periods, the *Arabidopsis* and *Solanum* plants were removed from the exposure conditions, photographed, and the fresh weight of plant shoots and total chlorophyll content were measured. Total chlorophyll content of plants exposed to *Trichoderma* VOCs was determined by submerging the shoot overnight in 1 ml of 80 % acetone in the dark at 4 °C. The total chlorophyll content (chlorophyll a and b) was calculated from the equation [(8.02)(A663) + (20.2)(A645)]*V*/1000 × *W*, where *V* is volume and *W* is plant fresh weight [24]. The chlorophyll data were expressed in relation to the fresh weight of the plant shoot. Five replicates were used per volatile exposure condition, and the experiments were repeated three times. Quantitative results were expressed as standard error of the mean and analyzed using R Statistical Software (version 3.2.3 Wooden Christmas Tree). One-way analysis of variance (ANOVA) and Student’s t test were performed for plant exposure quantitative data.

### CO_2_ assays

Total CO_2_ production by *Trichoderma* was measured using a CO_2_ meter (CO_2_Meter Inc. Osmond Beach, FL) at 8 h intervals for the duration of the experiment. Trapping experiments were performed in the plate-within-a-plate system where plants and fungi were grown in a shared atmosphere as described above. In separate experiments, a sterile cotton ball containing 3 ml of 0.1 M KOH was placed onto a sterile polypropylene cap (13 mm diameter). Then the cap containing the cotton ball was placed into an empty region of the plate containing the fungal culture. At the end of the exposure period, the cotton ball was fully dried and the dry weight of K_2_CO_3_ was obtained.

### VOC analysis by headspace GC–MS

For headspace volatile analysis, *Trichoderma* cultures were grown in a 500 ml glass flask containing 250 ml of MEA with a 16 h photoperiod at 27 ± 2 °C for 7 days. VOC capture and analysis were conducted as described previously using a purge and trap method [25]. Headspace samples taken from sterile MEA served as negative controls. The headspace of the flask was purged at 100 ml per min for 4 h. The VOCs were adsorbed on 6 cm Tenax columns (Scientific Instrument Services, Ringoes, NJ), recorded and analyzed with a Varian 3400 gas chromatograph (GC) mated to a Finnigan Mat 8230 mass spectrometer (MS). The GC was equipped with a 60 m, Equity-5 (SigmaAldrich Corp., St. Louis, MO) column: 0.32 mm diameter, 1 mm film thickness. The compounds were desorbed onto a −20 °C cryotrap with a TD-4 short path thermal desorption apparatus (Scientific Instrument Services, Ringoes, NJ). The GC conditions were: 10:1 split, helium carrier at 20 psi, oven temperature from −20 to 280 °C at 10 °C per min. The MS conditions were: positive ion mode, electron impact spectra at 70 eV. The MS of the peaks were determined by their scatter pattern. Internal standards (d-6 benzene, d-8 toluene, and d-8 naphthalene) were used to normalize the peak areas. The linear regression coefficient was used to calculate the concentrations in the samples from peak areas obtained in the chromatographs. Compounds were identified by comparison of spectra obtained from the *Trichoderma* samples with those from a reference library (NIST 08 Mass Spectra Library, National Institute of Standards and Technology). GC–MS analysis was conducted in triplicates for each strain.

## Results

### Strain specific volatile-mediated plant growth promotion

**Fig. 1:**
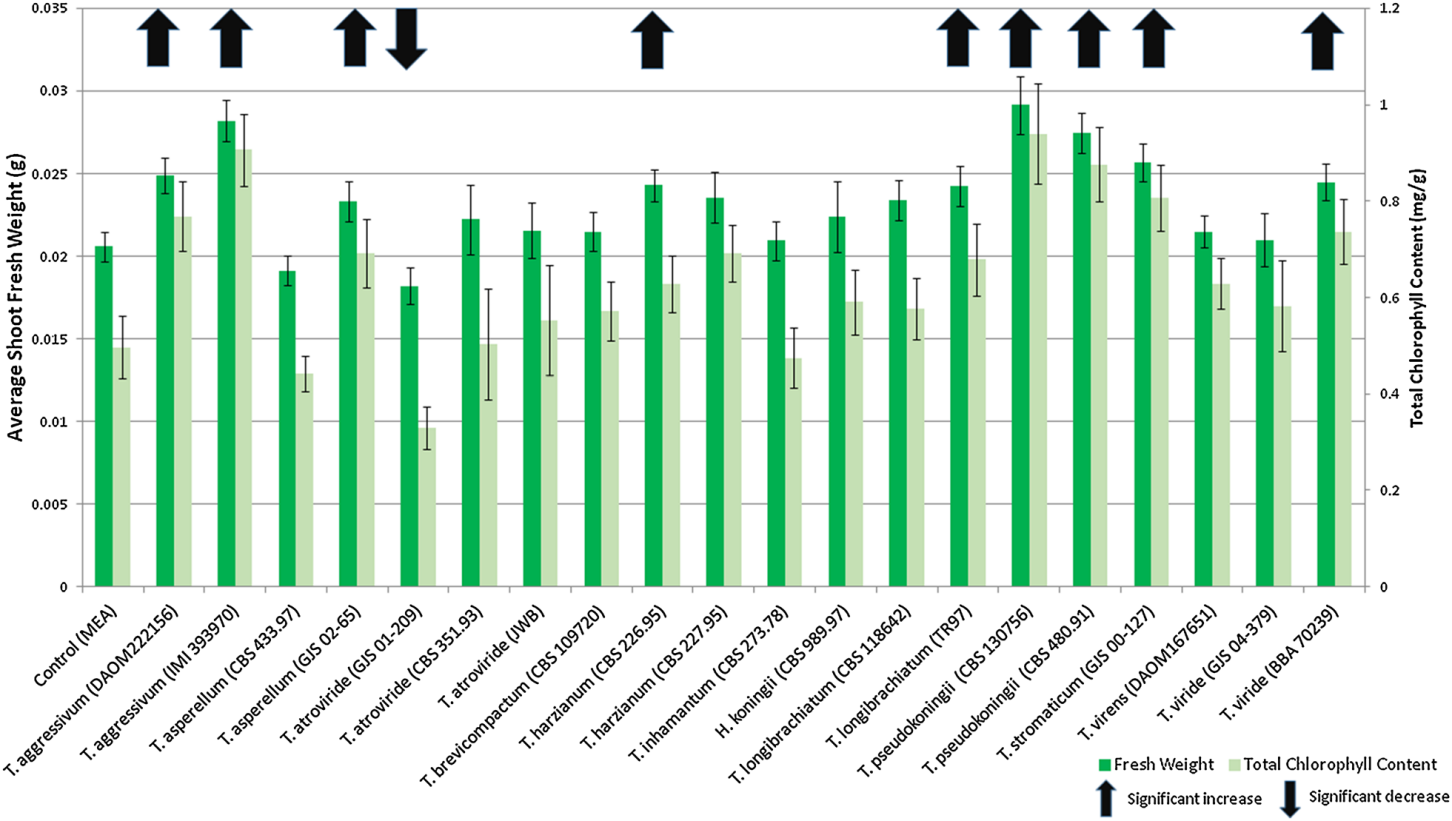
Average fresh weight and total chlorophyll content of *Arabidopsis thaliana* plants grown in a shared atmosphere with 20 different strains of *Trichoderma* for 14 days. Controls were grown without fungi. (n = 25, ANOVA P = 0.001)

**Fig. 2:**
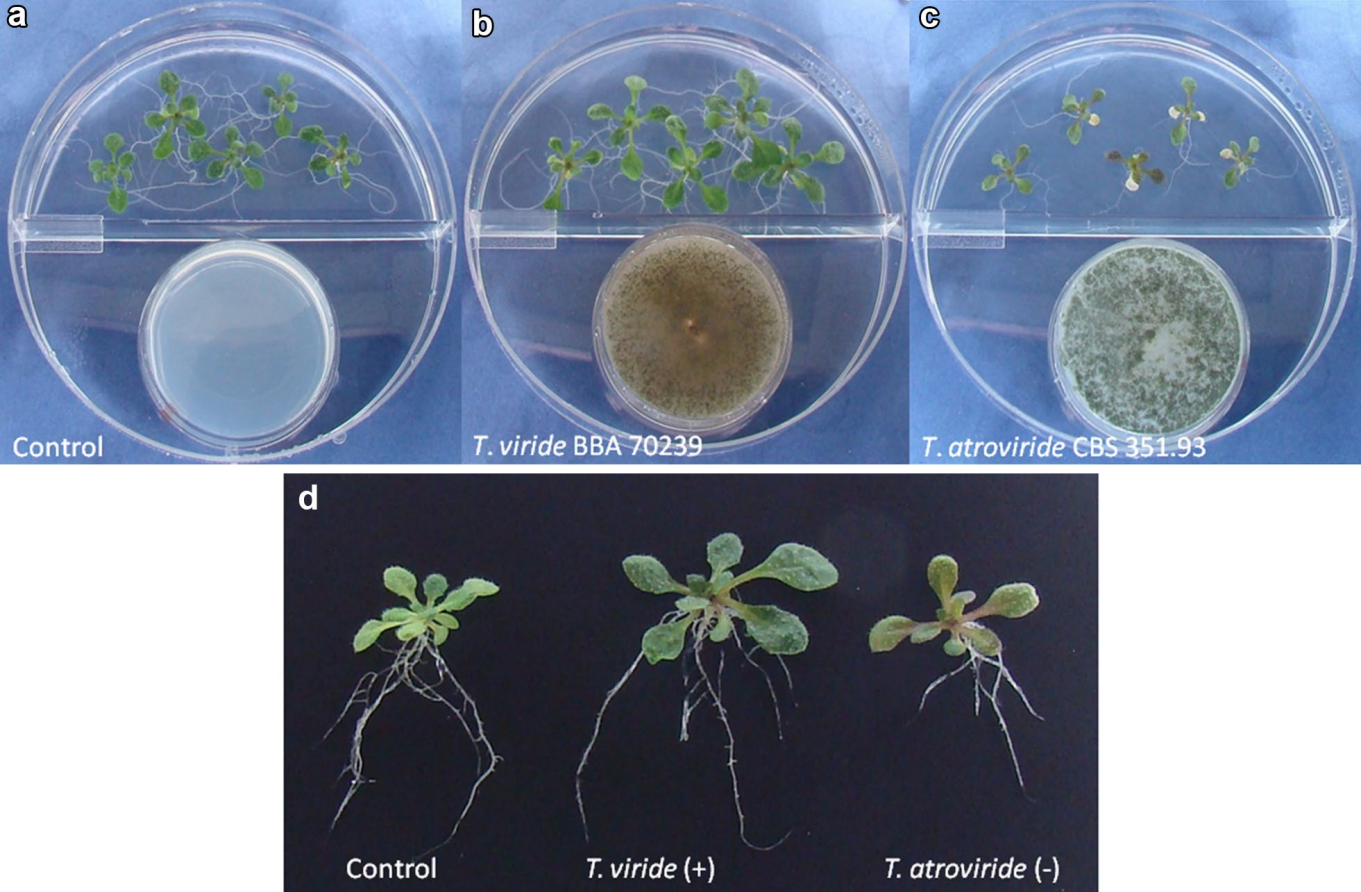
Growth of *Arabidopsis thaliana* in a shared atmosphere with *Trichoderma* for 14 days. **a** Control plants exposed to MEA medium, **b** plants exposed to *T. viride* (BBA 70239) are larger, **c** plants exposed to *T. atroviride* (CBS 351.93) are smaller, and **d** *Arabidopsis* plants removed from growth medium following 14-day *Trichoderma* volatile exposure

Twenty *Trichoderma* strains (Table 1) were screened. Following 14 days of exposure, plants were collected for measurement of fresh weight and total chlorophyll content of plant shoots (see Fig. 1). We defined a strain as growth promoting if both the plant fresh shoot weight and total chlorophyll content were significantly higher than controls. We identified nine strains representing six different species that promoted *Arabidopsis* growth: *T. aggressivum* (strains DAOM222156 and IMI 393970); *T. asperellum* (GJS 02-65); *T. harzianum* (CBS 226.95); *T. longibrachiatum* (strains CBS 118642 and TR97); *T. pseudokoningii* (strains CBS 480.91 and CBS 130756); and *T. viride* (GJS 04-379). Plant growth was enhanced by exposure to the nine strains including *T. viride* (BBA 70239) which is shown as a representative in Fig. 2b. The strongest volatile-mediated plant effects were observed in *T*. *aggressivum* (IMI 393970) with an increase of 37.1 % in fresh shoot weight and 82.5 % in chlorophyll and in *T. pseudokoningii* (CBS 130756) with an increase of 41.6 and 89.3 %, respectively. In contrast *T. atroviride* (CBS 01-209) emitted inhibitory VOCs, leading to small plants with a 13.1 % decrease in fresh shoot weight and 50.5 % decrease in chlorophyll. In addition to a reduction in fresh weight, we observed localized death in leaves (Fig. 2c). Half of the strains screened in this study did not alter the growth of *Arabidopsis* plants under our test conditions and looked similar to control plants (Fig. 2a).

We measured CO_2_ production by *Trichoderma* using a CO_2_ monitor and did not find a significant difference between CO_2_ production by the fungi and ambient air in our testing conditions. We observed similar CO_2_ levels between ambient and *Trichoderma* air ranging between 400 and 600 ppm of CO_2_ throughout the duration of our experiment. In addition, trapping *Trichoderma*-derived CO_2_ with 0.1 M KOH solution did not remove the observed volatile-induced beneficial effects. We observed a 41 % increase in chlorophyll for plants exposed to *Trichoderma* VOCs and KOH solution (0.68 mg/g) compared to negative control grown only in KOH (0.4 mg/g).

### The effects of *Trichoderma* volatiles on tomato shoot and root growths

**Fig. 3:**
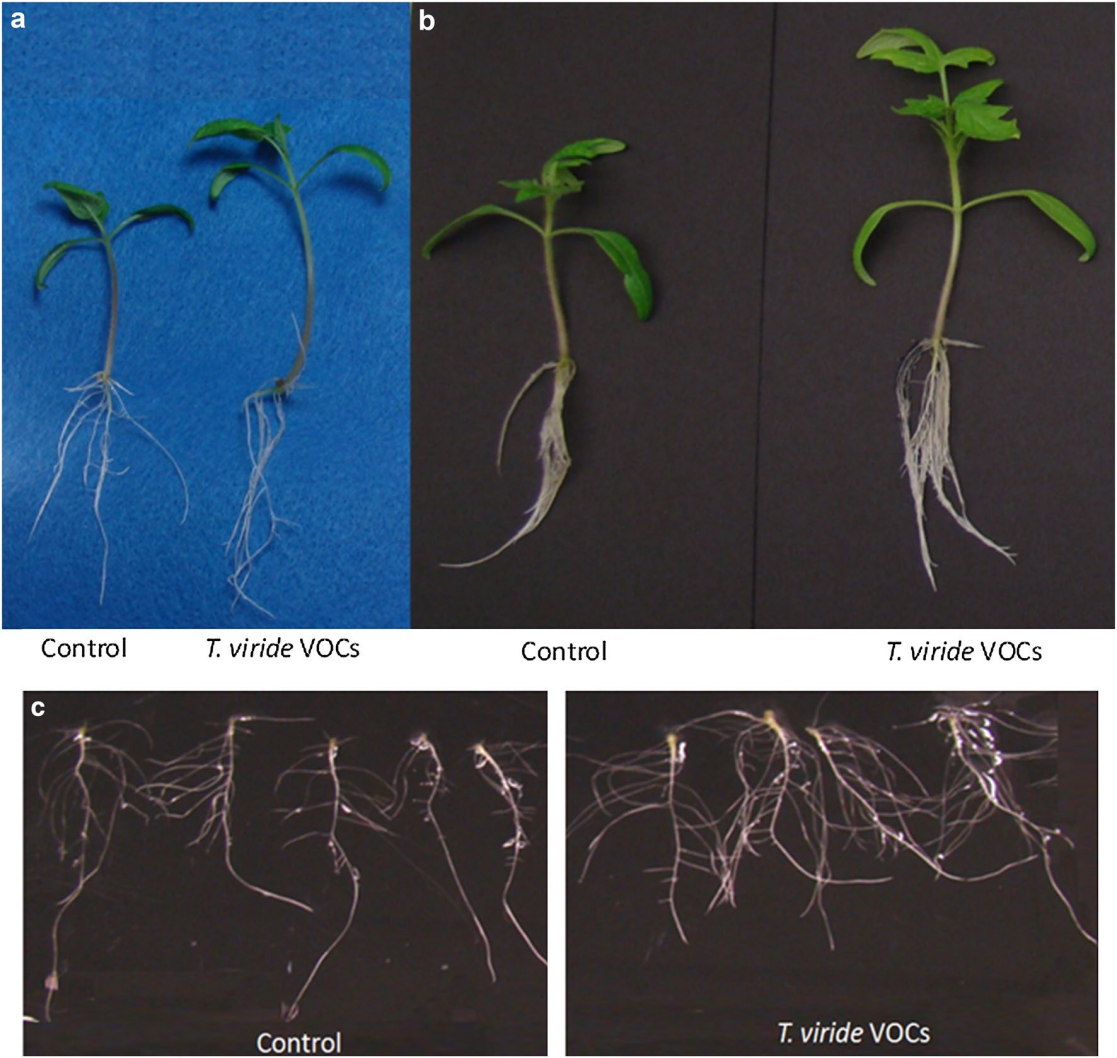
Tomato seedlings exposed to *T. viride* (BBA 70239) VOCs for **a** 14 days and **b** 21 days. **c** Roots of tomatoes exposed to *Trichoderma* VOCs for 21 days. Average fresh root weight of tomato seedlings exposed to *Trichoderma* VOCs for 21 days (0.135 ± 0.01 g) compared to controls (0.084 ± 0.018 g)

**Fig. 4:**
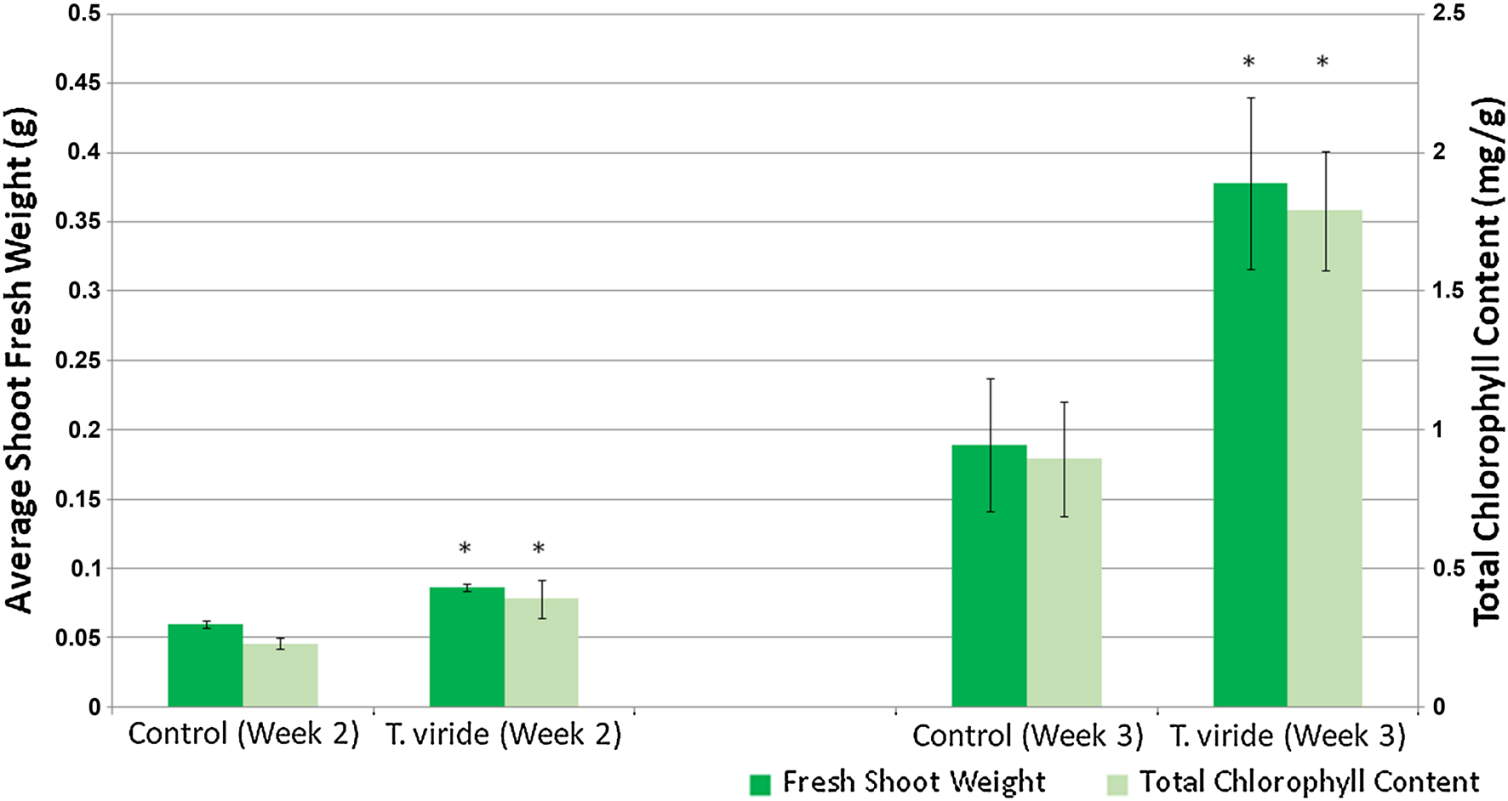
Shoot fresh weight and total chlorophyll content of tomato seedlings following 14- and 21-day-exposure to *T. viride* (BBA 70239) VOCs (n = 10, *P* = 0.01)

We selected the *Arabidopsis* growth promoting strain, *T*. *viride* (BBA 70239) and measured plant growth to assess its effects on tomato growth after 14 and 21 days of exposure. We selected this strain because it grew prolifically, sporulated readily, and was identified as a strain that did not produce the plant growth promoting compound, 6PP. Tomato seedlings exposed to *T. viride* VOCs were larger in size (Fig. 3), with increases in the lateral root development (Fig. 3c). The fresh root weight was 61.2 % greater than controls. Similarly, tomato plants exposed to *T. viride* VOCs for 14 days had a significant increase in biomass (41.2 %) and chlorophyll concentration (70.7 %). Extending the duration of volatile exposure to 21 days led to a larger increase in both tomato fresh weight (99.7 %) and chlorophyll (100.1 %) (Fig. 4).

### Identification of VOCs produced by *Trichoderma*

*Trichoderma* strains were grown separately on MEA for 7 days, and then the headspace was collected for 4 h and analyzed by GC–MS. *Trichoderma* strains differed in types and abundance of volatiles detected by headspace analysis. A total of 141 unique volatile compounds were detected at least twice per strain (Table 2). They encompassed hydrocarbons, alcohols, ketones, aldehydes, alkanes, alkenes, esters, aromatic compounds, heterocyclic compounds, and various terpenes. C8 and C10 compounds were dominant, making up 17.01 and 15.64 %, respectively, of the VOCs identified in this study. We also found unknown monoterpenes, sesquiterpenes, and tetraterpenes with molecular weights of 204, 222, 272 and 290 (data not presented). No terpenes were detected in headspace samples from MEA controls.

**Table 2:**
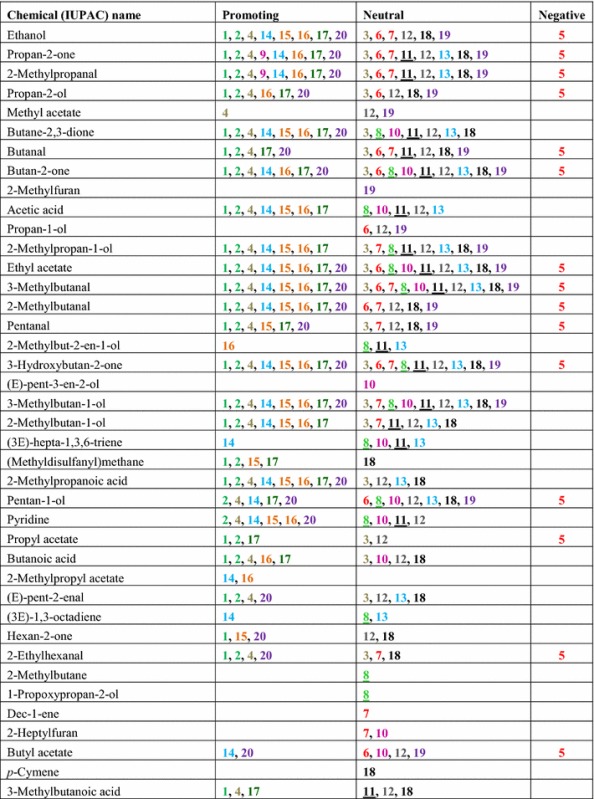

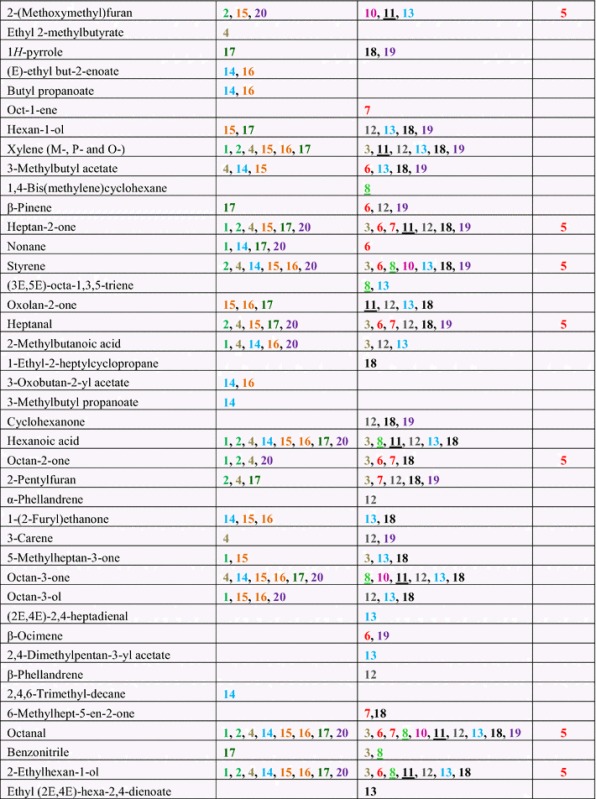

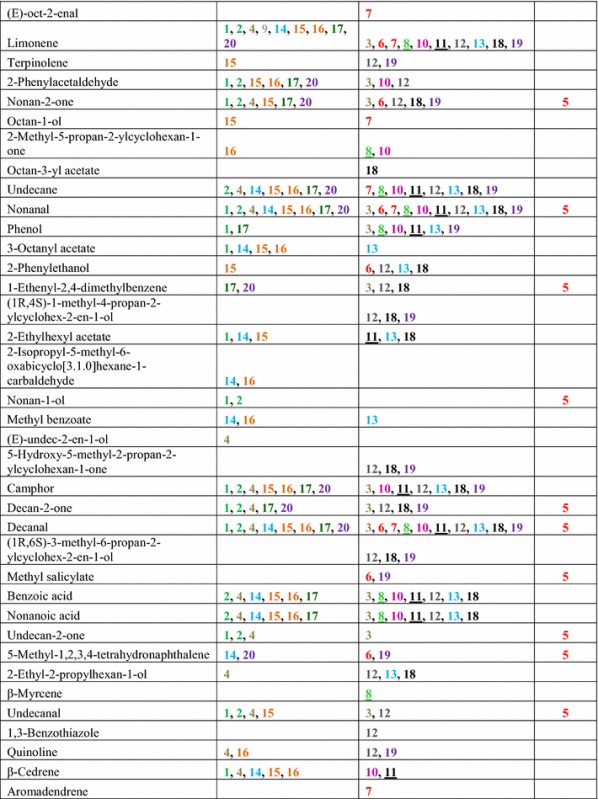

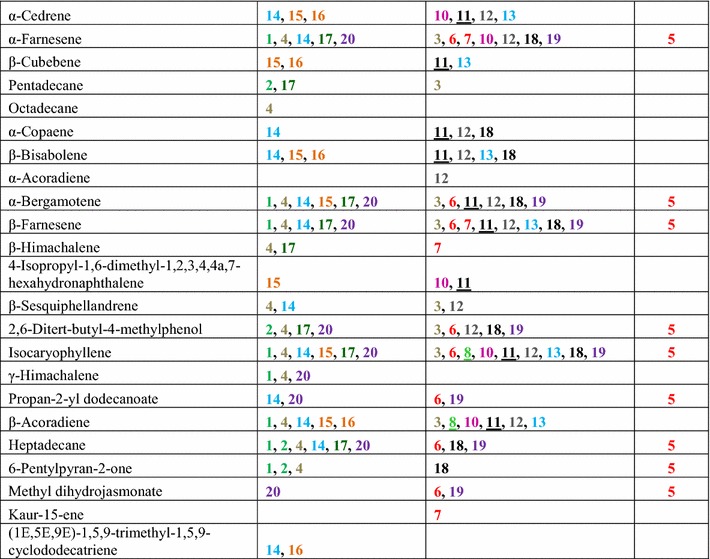
Headspace volatile collection of *Trichoderma* strains (100 ml/min, purge rate, 4 h, 1 µg Int. Std. by P&T-TD-GC–MS)

See Table 1 for strain number information. Same species are color coded

**Fig. 5:**
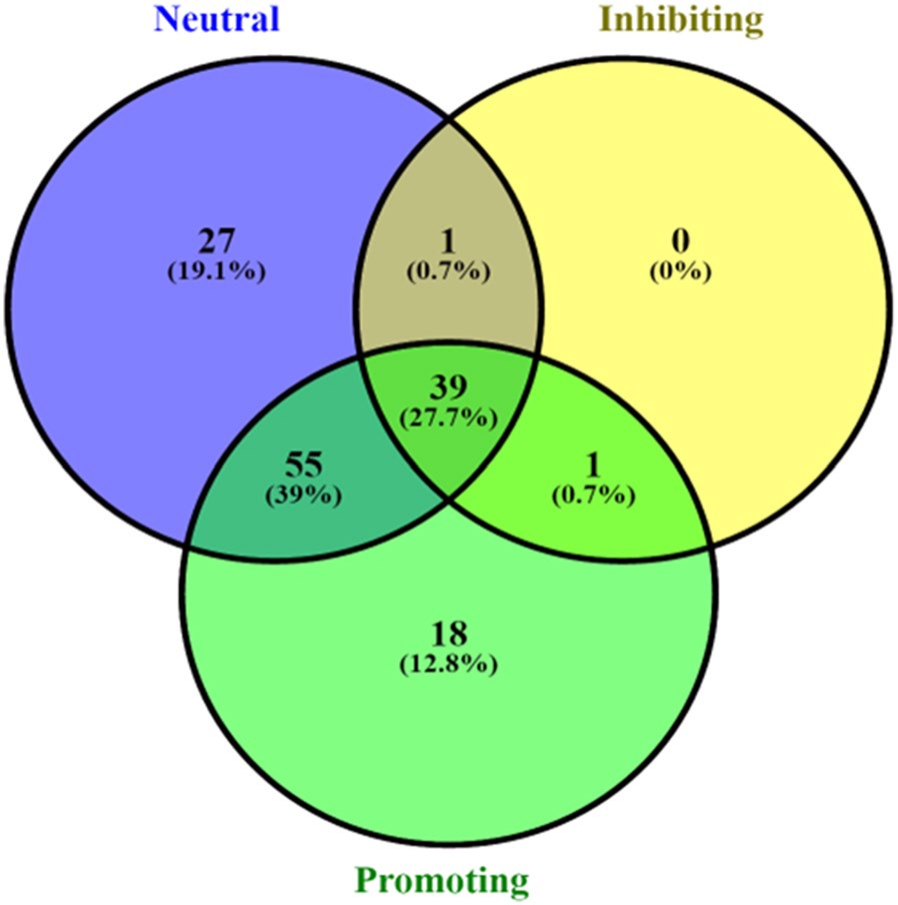
Comparison of *Trichoderma* volatile compounds identified by GC–MS. Compounds unique to *Trichoderma* strains that induced plant growth (*green*), inhibition (*yellow*), and no impact (*blue*)

A Venn diagram representing the complete volatile profile of *Trichoderma* strains identified by GC–MS is presented in Fig. 5. Positively acting (‘promoting’) strains lead to significant increases in overall plant biomass and chlorophyll while “inhibiting” strains lead to reduced size, biomass, and chlorophyll. “Neutral” strains did not significantly alter plant growth following volatile exposure. Approximately 27.7 % of the compounds identified were produced by 14 or more strains (Fig. 5; Table 2). Of these, only four compounds (3-methylbutanal, octanal, nonanal, and decanal) were found in all strains. Other commonly produced VOCs included acetoin and 2-butanone (found in 18 strains), 3-methyl-1-butanol (found in 17), and 2-methyl-1-propanol and acetone (both found in 16). Several terpenes were commonly identified including limonene (18 strains), β-caryophyllene (16 strains), β-farnesene (14 strains), and 2-norpinene (13 strains). The largest number of compounds (39 %) was shared between those that induced growth promotion and those that did not significantly impact growth.

One of the highest single concentrations of any volatile metabolite we identified in this study was 6-pentyl-2*H*-pyran-2-one [(6PP) 7559.45 ng/trap] produced by *T*. *atroviride* (GJS 01-209). This compound also was found in *T. aggressivum* (DAOM 222156 and IMI 393970), *T. asperellum* (GJS 02-65), and *T. virens* (DAOM 167651). Of these, only exposure to VOC mixtures from *T. aggressivum* and *T. asperellum* were associated with enhanced plant growth.

We found that 12.8 % of compounds identified were unique to plant growth promoting *Trichoderma* strains. They included ethyl 2-methylbutyrate and octadecane (*T*. *asperellum* GJS 02-65), 3-methylbutyl propanoate (*T*. *longibrachiatum* TR 97), and (2E,4E)-2,4-heptadienal (*T. longibrachiatum* CBS 118642). Compared to the neutral and inhibitory strains, plant growth promoting strains also produced a larger number of terpenes such as β-acoradiene, β-cubebene, β-cedrene, β-bisabolene, β-himachalene, and γ-himachalene. Compounds shared by growth promoting and neutral strains showed that only a minor portion of compounds (7 out of 56) were known to be produced from microorganisms while the rest are produced by both microorganisms and plants.

## Discussion

Volatile-mediated interactions between plants and microbes have been gaining increased attention in agriculture [26–29]. For example, VOCs have been proposed as biological control agents leading to the reduction of plant disease [30–33]. Furthermore, it is known that beneficial rhizosphere bacteria such as *Bacillus* produce VOCs that enhance plant growth [34–38]. The ability of soil fungi such as *Trichoderma* species to produce plant growth enhancing VOCs has been recognized only relatively recently [23–25]. In this study, we screened 20 *Trichoderma* biocontrol strains comprising 11 species and demonstrated that nine of these strains emitted VOC mixtures that significantly improved plant growth in *Arabidopsis* as measured by biomass, plant size, and chlorophyll concentration. Although *T*. *aggressivum* and *T*. *pseudokoningii* emitted VOCs that influenced *Arabidopsis* growth positively, we do not have a sufficient number of strains for each species to claim that the species as a whole can improve plant growth. It is known that increased CO_2_ levels associated with microbial growth in a Petri plate system can lead to plant growth promotion [39, 40]; however, we did not find significant differences in the level of CO_2_ in microhabitats containing *Trichoderma* and ambient air. Furthermore, sequestering *Trichoderma*-produced CO_2_ by absorption in the Petri system did not reduce the growth promotion observed.

In order to determine if we could duplicate the *Trichoderma* VOC response in a crop plant, we grew *S. lycopersicum* (tomato) seedlings in a shared atmosphere with the growth promoting strain, *T*. *viride* (BBA 70239). Exposed tomato plants also displayed significant increase in plant biomass, larger plant size, and increased lateral root development. Previously, we showed that *Trichoderma*-volatile induced growth promotion in *Arabidopsis* was dependent on the duration of exposure [25] and that early removal of the fungi lead to loss of growth promotion. In this study, we showed that a 3-week exposure had a greater proportional impact than a 2-week exposure. Moreover we observed an acceleration of the transition of vegetative phases. Prolonged exposure to microbial VOCs to trigger plant growth and development also have been observed in *Bacillus* [41, 42]. Together, these studies have shown the importance of the amount of volatile exposure time during different plant development phases [25, 41]. Based on the changes observed in the volatile-treated tomato seedlings, we believe that the VOC-induced acceleration of flower and fruit development warrants further investigation.

Which components of the volatile mixture emitted by growing *Trichoderma* strains cause the growth promotion effects? All microbial VOCs are found as complex mixtures and the volatile production is influenced by environmental conditions (i.e. nutrient content, microbial community composition, temperature, humidity, and pH), making it difficult to pinpoint either the effects of individual volatile molecules or their mechanisms of action [16, 25, 42–44]. We first looked at several individual compounds that have been identified in the literature as being stimulatory or inhibitory to plant growth. The compound 6-pentyl-2*H*-pyran-2-one (6PP), a lactone with a coconut-like odor, is commonly produced by *Trichoderma* and has been shown to both improve and inhibit plant growth and health at different concentrations [7, 23]. Although 6PP was produced by five *Trichoderma* strains in this study, the presence of 6PP was not unique to all growth promoting strains and was also found in strains that did not significantly impact plant growth. Previous reports showed that plants exposed to the volatile phase of 3-methyl-1-butanol, limonene, and acetoin lead to changes in plant size and chlorophyll concentration [34, 45] and we found that most of our strains produced 3-methyl-1-butanol, 2-methyl-1-propanol, limonene, β-farnesene, and β-caryophyllene, all known to be common microbial volatiles [17, 46–48]. However, since these compounds were found to be ubiquitous, they are not likely to be the cause of our observed growth promotion. Low concentrations of 1-hexanol, a truffle volatile, had a growth-promoting effect on *Arabidopsis* [38] while at higher concentrations, 1-hexanol inhibited plant growth [49] illustrating the importance of volatile concentration. The strain that caused reduction in plant growth did not produce detectable 1-hexanol in our study. Since the negatively impacting *T*. *atroviride* strain did not produce compounds unique to this strain, we suspect other factors such as concentration of individual compounds and changing volatile profile over time may have attributed to the negative effects observed in our study.

However, many of the compounds we identified are reported from plant research. Studies have shown that injured plants can influence the growth of neighboring plants through the release of certain plant VOCs that increase defense responses or growth [50, 51]. Several of the C8 compounds we found such as 3-octanol, 1-octanol, and 3-octanone have been characterized from wounded or infected plants [52–54]. Similarly, 3-carene, 2-methyl-1-butanol, butanoic acid, hexanoic acid, β-bisabolene, β-sesquiphellandrene, and β-acoradiene are released by plants under stress [55–58]. *Trichoderma* strains that increased plant growth and those that did not significantly alter plants both produced these VOCs. Similarly, a large number of diverse terpenes known to be produced by plants were detected in growth promoting strains. Finally, compounds unique to growth promoting strains (16 out of 18 compounds) were volatiles typically emitted from flowers and ripening fruits. Of these, octadecane, (2E,4E)-2,4-heptadienal, and (E)-pent-3-en-2-ol are emitted by plants under stress [59–61].

Even though we found several VOCs that have been studied as plant growth promoting compounds, these individual compounds alone do not explain the variation in growth induced by different *Trichoderma* strains. Fungi emit a large number of VOCs and the volatile profile changes as the fungi grow and mature. As with bacterial VOCs, the blend of volatiles produced and the time in plant development at which volatiles are applied, together, influence the outcome of the interactions [42]. Since we have only taken a single time point for our GC-MS analysis, we recognize that our analysis is a temporal “snap shot” that does not capture the full range of VOCs likely to have been produced by the growing *Trichoderma* during the course of the plant exposure experiments. There are not enough samples to be statistically absolute in the volatile profiles and the concentrations of individual compounds. Nevertheless, because we detected several compounds produced by *Trichoderma* strains that are well known plant metabolites produced by plants under stressful conditions, we hypothesize that the volatiles emitted by growth promoting *Trichoderma* strains are mimicking plant metabolites, providing plant cues that ultimately trigger growth changes.

## Conclusions

In conclusion*, A. thaliana* grown in the presence of fungal VOCs emitted by different species and strains of *Trichoderma* exhibited a range of endpoints that included increased plant size, neutral effects, or more rarely, growth inhibition. Plants exposed to *Trichoderma* strains were generally larger in size and greener in color. Only some of our growth promoting strains produced microbial VOCs known to enhance plant growth. Compounds such as 6-pentyl-2*H*-pyran-2-one were not common to all promoting strains. We found that biostimulatory strains tended to have a larger number of complex terpenes which may explain the variation in growth induced by different *Trichoderma* strains. It has been pointed out by Bailey and Melnick [62] that most *Trichoderma* research focuses on obtaining one candidate strain for formulation into an optimal biological control agent, with little work focused on the differences between strains. This study demonstrates that the blend of volatiles produced by actively growing fungi, strain-specific volatile profile, and volatile exposure time in plant development influence the outcome of volatile-mediated interactions.

## Abbreviations

VOCs: volatile organic compounds
6PP: 6-pentyl-2*H*-pyran-2-one
GC–MS: gas chromatography–mass spectrometry
PDA: potato dextrose agar
MEA: malt extract agar
MS: Murashige and Skoog
ANOVA: analysis of variance
GC: gas chromatograph
MS: mass spectrometer

## References

[1] Schuster A, Schmoll M (2010). Biology and biotechnology of *Trichoderma*. Appl Microbiol Biotechnol.

[2] Mukherjee PK, Horwitz BA, Singh US, Mukherjee M, Schmoll M, Mukherjee PK, Horwitz BA, Singh US, Mukherjee M, Schmoll M (2013). *Trichoderma* in agriculture, industry and medicine: an overview. *Trichoderma* biology and applications.

[3] Hoitink HAJ, Madden LV, Dorrance AE (2006). Systemic resistance induced by *Trichoderma* spp.: interactions between the host, the pathogen, the biocontrol agent, and soil organic matter quality. Phytopathology.

[4] Mathys J, De Cremer K, Timmermans P, Van Kerckhove S, Lievens B, Vanhaecke M, Cammue BP, De Coninck B (2012). Genome-wide characterization of ISR induced in *Arabidopsis thaliana* by *Trichoderma hamatum* T382 against *Botrytis cinerea* infection. Front Plant Sci.

[5] Howell CR, Harman GE, Kubicek CP (1998). The role of antibiosis in biocontrol. *Trichoderma* and *Gliocladium*.

[6] Contreras-Cornejo HA, Macías-Rodríguez L, Cortés-Penagos C, López-Bucio J (2009). *Trichoderma virens*, a plant beneficial fungus, enhances biomass production and promotes lateral root growth through an auxin-dependent mechanism in *Arabidopsis*. Plant Physiol.

[7] Harman GE, Howell CR, Viterbo A, Chet I, Lorito M (2004). *Trichoderma* species—opportunistic, avirulent plant symbionts. Nat Rev Microbiol.

[8] Szabo M, Csepregi K, Galber M, Fekete C (2012). Control plant-parasitic nematodes with *Trichoderma* species and nematode-trapping fungi: the role of chi18-5 and chi18-12 genes in nematode egg-parasitism. Biol Control.

[9] Singh PC, Nautiyal CS (2012). A novel method to prepare concentrated conidial biomass formulation of *Trichoderma harzianum* for seed application. J Appl Microbiol.

[10] Gillespie AT, Moorhouse ER, Whipps JM, Lumsden RD (1989). The use of fungi to control pests of agricultural and horticultural importance. Biotechnology of fungi for improving plant growth.

[11] Mendoza-Mendoza A, Steyaert J, Nieto-Jacobo MF, Holyoake A, Braithwaite M, Stewart A (2015). Identification of growth stage molecular markers in *Trichoderma* sp. ‘atroviride type B’ and their potential application in monitoring fungal growth and development in soil. Microbiology.

[12] Chaparro AP, Carvajal LH, Orduz S (2011). Fungicide tolerance of *Trichoderma asperelloides* and *T. harzianum* strains. Agric Sci.

[13] Mathivanan N, Prabavathy VR, Vijayanandraj VR, Karlovsky P (2008). The effect of fungal secondary metabolites on bacterial and fungal pathogens. Secondary metabolites in soil ecology.

[14] Mukherjee PK, Horwitz BA, Kenerley CM (2012). Secondary metabolism in *Trichoderma*—a genomic perspective. Microbiology.

[15] Engelberth J, Koch T, Schüler G, Bachmann N, Rechtenbach J, Boland W (2001). Ion channel-forming alamethicin is a potent elicitor of volatile biosynthesis and tendril coiling. Cross talk between jasmonate and salicylate signaling in lima bean. Plant Physiol.

[16] Insam H, Seewald SA (2010). Volatile organic compounds (VOCs) in soils. Biol Fertil Soils.

[17] Korpi A, Jarnberg J, Pasanen AL (2009). Microbial volatile organic compounds. Crit Rev Toxicol.

[18] Lemfack MC, Nickel J, Dunkel M, Preissner R, Piechulla B (2014). VOC: a database of microbial volatiles. Nucleic Acids Res.

[19] Kesselmeier J, Staudt M (1999). Biogenic volatile organic compounds (VOC): an overview on emission, physiology and ecology. J Atmos Chem.

[20] Dudareva N, Klempien A, Muhlemann JK, Kaplan I (2013). Biosynthesis, function and metabolic engineering of plant volatile organic compounds. New Phytol.

[21] Collins RP, Halim AF (1972). Characterization of the major aroma constituent of the fungus *Trichoderma viride* (Pers.). J Agric Food Chem.

[22] Parker SR, Hill RA, Cutler HG, Cutler HG, Cutler SJ (1999). Spectrum of activity of antifungal natural products and their analogs. Biologically active natural products: agrochemicals.

[23] Vinale F, Sivasithamparam K, Ghisalberti EL, Marra R, Woo SL, Lorito M (2008). *Trichoderma*-plant pathogens interactions. Soil Biol Biochem.

[24] Hung R, Lee S, Bennett JW (2013). *Arabidopsis thaliana* as a model system for testing the effects of *Trichoderma* volatile organic compounds. Fungal Ecol.

[25] Lee S, Hung R, Yap M, Bennett JW (2015). Age matters: the effects of volatile organic compounds emitted by *Trichoderma atroviride* on plant growth. Arch Microbiol.

[26] Schulz S, Dickschat JS (2007). Bacterial volatiles: the smell of small organisms. Nat Prod Rep.

[27] Piechulla B, Degenhardt J (2014). The emerging importance of microbial volatile organic compounds. Plant Cell Environ.

[28] Bitas V, Kim H-S, Bennett JW, Kang S (2013). Sniffing on microbes: diverse roles of microbial volatile organic compounds in plant health. Mol Plant Microbe Interact J.

[29] Junker RR, Tholl D (2013). Volatile organic compound mediated interactions at the plant–microbe interface. J Chem Ecol.

[30] Wheatley R, Hackett C, Bruce A, Kundzewicz A (1997). Effect of substrate composition on production of volatile organic compounds from *Trichoderma* spp. inhibitory to wood decay fungi. Int Biodeterior Biodegrad.

[31] Bruce A, Wheatley RE, Humphris SN, Hackett CA, Florence MEJ (2000). Production of volatile organic compounds by *Trichoderma* in media containing different amino acids and their effect on selected wood decay fungi. Holzforschung.

[32] Aguero LEM, Alvarado R, Martinez A, Dorta B (2008). Inhibition of *Aspergillus flavus* growth and aflatoxin B1 production in stored maize grains exposed to volatile compounds of *Trichoderma harzianum* Rifai. Interciencia.

[33] Campos VP, Pinho RSC, de Freire ES (2010). Volatiles produced by interacting microorganisms potentially useful for the control of plant pathogens. Ciencia e Agrotec Lavras.

[34] Ryu CM, Farag MA, Hu CH, Reddy MS, Wie HX, Pare PW, Kloepper JW (2003). Bacterial volatiles promote growth of *Arabidopsis*. Proc Nat Acad Sci.

[35] Ryu CM, Farag MA, Hu CH, Reddy MS, Kloepeer JW, Pare PW (2004). Bacterial volatiles induce systemic resistance in *Arabidopsis*. Plant Physiol.

[36] Lugtenberg B, Kamilova F (2009). Plant-growth-promoting rhizobacteria. Annu Rev Microbiol.

[37] Cortes-Barco AM, Goodwin PH, Hsiang T (2010). Comparison of induced resistance activated by benzothiadiazole, (2R, 3R)-butanediol and an isoparaffin mixture against anthracnose of *Nicotiana benthamiana*. Plant Pathol.

[38] Blom D, Fabbri C, Connor EC, Schiestl FP, Klauser DR, Boller T, Eberl L, Weisskopf L (2011). Production of plant growth modulating volatiles is widespread among rhizosphere bacteria and strongly depends on culture conditions. Environ Microbiol.

[39] Kai M, Piechulla B (2009). Plant growth promotion due to rhizobacterial volatiles—an effect of CO_2_?. FEBS Lett.

[40] Yang J, Kloepper JW, Ryu CM (2009). Rhizosphere bacteria help plants tolerate abiotic stress. Trends Plant Sci.

[41] Xie X, Zhang H, Paré PW (2009). Sustained growth promotion in *Arabidopsis* with long-term exposure to the beneficial soil bacterium *Bacillus subtilis* (GB03). Plant Signal Behav.

[42] Bailly A, Weisskopf L (2012). The modulating effect of bacterial volatiles on plant growth. Plant Signal Behav.

[43] McNeal KS, Herbert BE (2009). Volatile organic metabolites as indicators of soil microbial activity and community composition shifts. Soil Sci Soc Am J.

[44] Polizzi V, Adams A, Picco AM, Adriaens E, Lenoir J, Peteghem CV, Saegar SD, Kimpe ND (2011). Influence of environmental conditions on production of volatiles by *Trichoderma atroviride* in relation with sick building syndrome. Build Environ.

[45] Hung R, Lee S, Bennett JW (2015). Fungal volatile organic compounds and their role in ecosystems. Appl Microbiol Biotechnol.

[46] Pasanen P, Korpi A, Kalliokosi P, Pasanen AL (1997). Growth and volatile metabolite production of *Aspergillus versicolor* in house dust. Environ Int.

[47] Fiedler K, Schutz E, Geh S (2001). Detection of microbial volatile organic compounds (MVOCs) produced by moulds on various materials. Int J Hyg Environ Health.

[48] Jelen H, Blaszczyk L, Jerzy C, Rogowicz K, Strakowska J (2014). Formation of 6-*n*-pentyl-2*H*-pyran-2-one (6-PAP) and other volatiles by different *Trichoderma* species. Mycol Prog.

[49] Splivallo R, Novero M, Bertea CM, Bossi S, Bonfante P (2007). Truffle volatiles inhibit growth and induce an oxidative burst in *Arabidopsis thaliana*. New Phytol.

[50] Pierik R, Mommer L, Voesenek LACJ (2013). Molecular mechanisms of plant competition: neighbor detection and response strategies. Funct Ecol.

[51] Blande JD, Holopainen JK, Niinemets Ü (2014). Plant volatiles in a polluted atmosphere: stress response and signal degradation. Plant Cell Environ.

[52] Cardoza YJ, Alborn HT, Tumlinson JH (2002). In vivo volatile emissions from peanut plants induced by simultaneous fungal infection and insect damage. J Chem Ecol.

[53] Tabata J, De Moraes CM, Hescher MC (2011). Olfactory cues from plants infected by powdery mildew guide foraging by a mycophagous ladybird beetle. PLoS One.

[54] Mohanta TK, Occhipinti A, Atsbaha Zebelo S, Foti M, Fliegmann J, Bossi S, Maffei ME, Bertea CM (2012). *Ginkgo biloba* responds to herbivory by activating early signaling and direct defenses. PLoS One.

[55] Snoeren TAL, Kappers IF, Broekgaarden C, Mumm R, Dicke M, Bouwmeester HJ (2010). Natural variation of herbivore-induced volatiles in *Arabidopsis thaliana*. J Exp Bot.

[56] Cai L, Koziel JA, O’Neal ME (2015). Studying plant–insect interactions with solid phase microextraction: screening for airborne volatile emissions response of soybeans to the soybean aphid, *Aphis glycines* Matsumura (Hemiptera:Aphididae). Chromatography.

[57] Joutsensaari J, Yli-Pirila P, Korhonen H, Arola A, Blande JD, Heijari J, Kivimaenpaa M, Mikkonen S, Hao L, Miettinen P, Lyytikainen-Saarenmaa P, Faiola CL, Laaksonen A, Holopainen JK (2015). Biotic stress accelerates formation of climate-relevant aerosols in boreal forests. Atmos Chem Phys.

[58] Llorens E, Camañes G, Lapeña L, García-Agustín P (2016). Priming by hexanoic acid induce activation of mevalonic and linolenic pathways and promotes the emission of plant volatiles. Front Plant Sci.

[59] Reisenman CE, Riffell JA, Duffy K, Pesque A, Mikles D, Goodwin B (2012). Species-specific effects of herbivory on the oviposition behavior of the moth *Manduca sexta*. J Chem Ecol.

[60] Jardine KJ, Meyers K, Abrell L, Alves EG, Yanez Serrano AM, Kesselmeier J, Karl T, Guenther A, Chambers JQ, Vickers C (2013). Emissions of putative isoprene oxidation products from mango branches under abiotic stress. J Exp Bot.

[61] Tanaka K, Taniguchi S, Tamaoki D, Yoshitomi K, Akimitsu K, Gomi K (2014). Multiple roles of plant volatiles in jasmonate-induced defense response in rice. Plant Signal Behav.

[62] Bailey BA, Melnick RL, Mukherjee PK, Horwitz BA, Singh US, Mukherjee M, Schmoll M (2013). The endophytic *Trichoderma*. *Trichoderma*: biology and applications.

